# *Lactobacillus plantarum* 45 activates SHP2 through inhibition of oxidative stress to regulate osteoblast and osteoclast differentiation

**DOI:** 10.18632/aging.205708

**Published:** 2024-04-03

**Authors:** Yaming Yang, Zheng Yan, Qi Xie, Yong Wang, Zhiying Liu, Min Lei

**Affiliations:** 1 Department of Clinical Nutrition, The Third Hospital of Hebei Medical University, Shijiazhuang, Hebei 050051, China; 2 School of Public Health, Hebei Medical University, Shijiazhuang, Hebei 050017, China; 3 Department of Nutrition, The Fourth Hospital of Hebei Medical University, Shijiazhuang, Hebei 050011, China; 4 Department of Research, The Third Hospital of Hebei Medical University, Shijiazhuang, Hebei 050051, China

**Keywords:** LP45, osteoporosis, SHP2, oxidative stress, osteoblast, osteoclast, inflammation

## Abstract

Background: The purpose of this study is to observe LP45 (*Lactobacillus plantarum* 45) to investigate the mechanism by which LP45 attenuates oxidative stress-induced damage and regulates the osteoblast-osteoclast balance.

Materials and Methods: The oxidative stress level and osteoblast- and osteoclast-related proteins were detected by immunofluorescence staining, Western blotting, ROS fluorescent probe and ELISA. Osteoblast cell proliferation capacity was determined by the CCK-8 assay. X-ray observation and HE staining were used to detect the effect of LP45 on osteoporosis.

Results: The expression level of SHP2 and Src was significantly increased, and the expression levels of NOX4, P22, P47, IL-1β, NLRP3, IRF3, RANK, β-catenin and INF-β were inhibited in LP45 group and LPS + LP45 group as compared to those in LPS group. Compared with that in LPS group, the concentration of SOD was increased and the concentration of MDA was decreased in LPS + LP45 group. The protein expressions of OPG, RANKL, RUNX3, RANK and β-catenin in LP45 group and LPS + LP45 group increased. The protein expressions of NF-κB, CREB and AP-1 in LP45 group and LPS + LP45 group decreased significantly. The results were also confirmed by immunofluorescence staining and ROS fluorescent probe. X-ray observation and HE staining showed that LP45 could inhibit the progression of osteoporosis.

Conclusion: LP45 can exert its antioxidant effect by inhibiting the production of oxidative stress to activate the SHP2 signaling pathway, thus promoting osteoblast differentiation and repressing osteoclast formation to maintain bone homeostasis and improve bone metabolism.

## INTRODUCTION

Osteoporosis is a systemic skeletal disease that involves bone tissues, in which the bone mass is reduced, resulting in increased bone fragility and heightened risk of fractures or even death [1]. The balance of bone formation and degradation is essential for maintaining normal skeletal homeostasis, while an imbalance between the functioning of osteoblasts and osteoclasts may cause skeletal diseases of varying severities, the most common among which is osteoporosis [2]. Bone remodeling is a tightly coupled process involving osteoclasts and osteoblasts. Osteoclasts are multinucleated cells that inhibit H, Cl, cathepsin K (CTSK), and matrix metalloproteinases (MMPs) by activating macrophage colony-stimulating factor (M-CSF) and nuclear cytokine κB receptor activator ligand (RANKL). [3–5]. Normally, intracellular primary reactive oxygen species (ROS) are crucial players in signaling pathways, inflammatory responses, cell proliferation and apoptosis, and tissue repair [6]. A large body of literature has identified oxidative stress as a causative factor in osteoporosis. Osteoclast differentiation is influenced by oxidative stress. Oxidative stress can induce osteoblast apoptosis and reduce osteoblast activity, and in the occurrence and progression of osteoporosis, it is a principal factor that causes the imbalance of bone homeostasis [7, 8]. It is proposed that oxidative stress can increase osteoclast production and osteoblast and osteocyte apoptosis, decrease osteoprogenitor differentiation to osteoblast lineages, and reduce osteoblast activity [9]. As such, inhibition of oxidative stress may be a linchpin in preventing or delaying osteoporosis.

Healthy intestinal flora is closely associated with bone homeostasis [10]. To be specific, intestinal flora can influence host bone homeostasis by mediating immune system functions to modulate bone metabolism [11], and intestinal flora disorders may enhance osteoclast activity, leading to the occurrence and progression of osteoporosis [12]. As active substances, probiotics have been proven to possess antioxidant properties and also to repress the proliferation of pathogenic microorganisms and reduce the production of lipopolysaccharide (LPS) and inflammatory cytokines through several mechanisms, such as increasing antibacterial properties via secretion of antibacterial metabolites and transformation of host substances, competing for energy substances, and cell contact-dependent specific killing [13]. Previous studies have indicated that probiotics are able to diminish bone loss, restrain osteoblast senescence, reduce bone resorption by osteoclasts, and elevate the expression of bone alkaline phosphatases [14, 15]. For instance, *Lactobacillus plantarum* (LP) has been validated to influence immune-related skeletal health by modulating the expressions of pro-inflammatory cytokines and markers associated with bone metabolism [16]. Probiotics may therefore play a positive role in the prevention and treatment of osteoporosis.

Src homology region 2 domain-containing protein tyrosine phosphatase-2 (SHP2) is a protein tyrosine phosphatase encoded by PTPN11, and its role in signal transduction requires multiple different cell surface receptors [17]. Evidence indicates that SHP2 has the ability to regulate a series of signaling pathways evoked by fibroblast growth factors, and its expression is notably raised under anti-oxidative stress [18], which is important for osteoblast and osteoclast differentiation, but its underlying molecular and cellular mechanisms are still obscure [19]. Previous studies have shown that macrophages secrete and transfer certain functional proteins and RNAs to influence many types of cells, such as fibroblasts and epithelial cells [20, 21], while its effects on osteoblasts and osteoclasts need to be explored in depth. Taken together, whether LP45 exerts antioxidant effects to regulate bone homeostasis through SHP2 by modulating macrophage-derived exosomes remains inconclusive, and the specific mechanism of their interactions needs to be clarified. This study reveals that LP45 exerts its anti-oxidative stress effect to activate SHP2 and thus promote osteoblast differentiation and repress osteoclast formation.

## MATERIALS AND METHODS

### Cells and reagents

Mouse pre-cranial osteoblast cell line (MC3T3-E1 cells) and mouse monocyte macrophage (RAW264.7 cells) were provided by Wuhan Procell Co., Ltd. (China). Reagents used in this study included freeze-dried bacterial powder of LP45 (Hebei Inatural Biotech Co., Ltd., China), cell culture medium (Dulbecco’s Modified Eagle Medium (DMEM), Sangon Biotech (Shanghai) Co., Ltd., China), fetal bovine serum (FBS, Sangon Biotech (Shanghai) Co., Ltd.), Transwell cell culture chambers (Shanghai Cuihua Biotechnology Co., Ltd.), LPS (Sigma-Aldrich, St. Louis, MO, USA), antibiotics (Shanghai Aladdin Biochemical Technology Co., Ltd., China), and prednisone acetate (Cisen Pharmaceutical Co., Ltd., Shandong, China).

### Cell culture and grouping

#### LP45 culture

LP45 was purchased from Hebei Yiran Biotechnology Co., Ltd. (China). LP45 was cultured in MRS medium at a constant temperature of 37°C. Then it grew in an incubator and passed three consecutive times, and after adjusting the bacterial solution concentration, the third generation of cells grew for 18 h to restore strain viability. The supernatant from LP45 was obtained by filtration (0.22 μm pore size), and LP45 was centrifuged at 4000 rpm at 4°C for 30 min. The LP45 concentration was adjusted to 1 × 10^8^ CFU/mL using a cell counter for later use.

#### Macrophage culture and grouping

Macrophage RAW264.7 cells were cultured in high-glucose DMEM (HDMEM, HyClone, Logan, UT, USA) supplemented with 10% FBS and antibiotics. RAW264.7 cells were passaged for 3 generations, and then they were inoculated into 96-well plates at a density of 2 × 10^5^ cells/mL (100 μL/well). After 16–20 h of cell adhesion, the fresh culture medium was replaced. Cells were divided into four groups: LPS group (HDMEM containing LPS (10 μg/mL) was added to the continuous medium for 12 h), LP45 group (HDMEM containing LP45 (1 × 10^8^ CFU/mL) was added to the continuous medium for 12 h), LPS + LP45 group (after adding HDMEM containing LPS (10 μg/mL) for 6 h, LP45 (1 × 10^8^ CFU/mL) was added for 6 h), and blank control group (addition of phosphate-buffered saline (PBS) as a blank control).

#### Osteoblast and osteoclast culture

MC3T3-E1 cells were seeded in 6-well cell culture plates at a density of 1 × 10^6^ cells/well and cultured in Minimum Essential Medium α (α-MEM) containing osteogenic inducer (50 μg/mL ascorbic acid, 10 mmol/L β-sodium glycerophosphate, and 10–8 mol/mL dexamethasone), 10% FBS and 1% penicillin-streptomycin in an incubator with 5% CO_2_ at 37°C. Macrophage RAW264.7 cells were then transferred to MEM containing 10% FBS. Next, bone marrow cells were filtered using a 70-μm filter and plated in a T75 culture flask containing MEM + 10% FBS + 10 ng/mL M-CSF medium. After 24 h of culture in an incubator with 5% CO_2_ at 37°C, the cells were harvested from the supernatant and plated in MEM + 10% FBS + 30 ng/mL M-CSF medium at a density of 2 × 10^5^ cells/mL, and after 48 h, cell differentiation was induced by adding 100 ng/mL RANKL into the medium for 14 days.

#### Cell co-culture system and grouping

The cell co-culture system was constructed using Transwell chambers. Specifically, after washing with PBS, mouse osteoblasts (MC3T3-E1 cells) and osteoclasts were digested with trypsin and inoculated and cultured in a 6-well Transwell lower chamber at a density of 5 × 10^4^ cells/mL separately. Next, mouse RAW264.7 cells were digested with trypsin and inoculated and cultured in another 6-well Transwell upper chamber at a density of 2.5 × 10^4^ cells/mL. Then osteoblasts and osteoclasts were pretreated with glucocorticoids (1 μmol/L), and the macrophage-osteoblast culture system and macrophage-osteoclast culture system were each divided into four groups: LPS group (10 μg/mL LPS), LP45 group (1 × 10^8^ CFU/mL LP45), LPS + LP45 group (10 μg/mL LPS + 1 × 10^8^ CFU/mL LP45), and blank control group (addition of PBS as a blank control). Transiently transfect macrophages with siRNA-NC and siRNA-SHP2 using Lipofectamine 2000 (Invitrogen Life Technologies, Carlsbad, CA, USA) according to the manufacturer’s instructions. Transfection for 48 h. siRNA-SHP2: 5′-TCCCGACCCTTATCGTACGATCTAATAAATTCAAGAGATTCTACTATCTTACTTATTATCTATTT-3′; siRNA-NC: 5′-TCCCTTCTCCGAACGTGTCACGTTTCAGAGAACGTGACGTTCGGAGAATT-3′.

### CCK-8 cell proliferation capacity assay

After MC3T3-E1 cells were grown to 3 passages, the cell concentration was adjusted to 2105 cells/mL, and 100 μL of cell fluid per well was docked at 96. In well plates, after 16–20 h of cell adhesion, fresh culture media was replaced and 10 μL of the batch fermentation supernatant was seeded per well at 96-well plates, seeded with 10 μL of cell culture medium as a blank group and incubated in a 5% CO_2_ incubator at 37°C for 12 h before reference. CCK-8 kit cell proliferation was measured by the instructions: 10 μL of CCK-8 solution was added to each well in the cell incubator. Incubation for 0, 24, 48 h and the OD was measured at 450 nm.

### Western blotting

First, RAW264.7 cells and MC3T3-E1 cells were taken from each group and lysed. After boiling, sonication and centrifugation, the supernatant was harvested to obtain protein samples, of which the concentrations were quantified. BCA protein detection kit was used (Beijing Solar Biotechnology Co., Ltd., Beijing, China). Then, 20 μg of protein was treated with SDS-PAGE, transferred to the cell membrane, and sealed with 5% skim milk for one hour. Subsequently, 20 μg of proteins were subjected to SDS-PAGE, transferred onto membranes, and blocked with 5% skimmed milk for 1 h. After that, macrophage RAW264.7 protein samples were incubated with primary antibodies against nicotinamide adenine dinucleotide phosphate oxidase 4 (NOX4) (Abcam, UK, ab154244, 1:1000), P22 (Abcam, ab191512, 1:500), P47 (Abcam, ab308256, 1:1000), Src (Abcam, ab133283, 1:1000), interleukin-1 beta (IL-1β) (Abcam, ab254360, 1:1000), nucleotide-binding oligomerization domain-like receptor protein 3 (NLRP3) (Abcam, ab263899, 1:1000), interferon regulatory factor 3 (IRF3) (Abcam, ab68481, 1:1000), interferon-β (INF-β) (Abcam, ab313885, 1:1000) and glyceraldehyde-3-phosphate dehydrogenase (GAPDH) (Abcam, ab9485, 1:2500), while osteoblast (MC3T3-E1 cells) and osteoclast protein samples were incubated with primary antibodies against osteoprotegerin (OPG) (Abcam, ab183910, 1:1000), RANKL (Abcam, ab9957, 1:1000), runt-related transcription factor 3 (RUNX3) (Cell Signaling Technology, USA, ab154244, 1:1000), RANK (Abcam, ab305233, 1:1000), β-catenin (Abcam, ab32572, 1:5000) nuclear factor-kappa B (NF-κB) (Abcam, ab207297, 1:1000), cAMP-responsive element-binding protein (CREB) (Abcam, ab32515, 1:1000), activator protein-1 (AP-1) (Abcam, ab230273, 1:1000) and GAPDH (Abcam, ab9485, 1:2500), overnight at 4°C. The next day, the membranes were washed and incubated with secondary antibodies at room temperature for 1 h. After washing, the membranes were visualized using ECL reagents, followed by semi-quantitative analysis using ImageJ 1.52 software.

### Immunofluorescence staining

The expression of RANKL on the osteoblast surface was detected by immunofluorescence technique. Osteoblasts were fixed with 4% paraformaldehyde for 30 min, washed three times with PBS, blocked with 5% bovine serum albumin for 1 h, and then incubated with RANKL antibody (Abcam, ab65024, 1:500) overnight at 4°C. After washing, fluorescent goat anti-rabbit secondary antibody IgG (Abcam, ab150077, 1:500) was added for incubation in the dark for 2 h. After washing, anti-fluorescence quenching sealing agent DAPI was added to seal the membrane, and images were observed and photographed under a fluorescence microscope.

### ROS fluorescent probe

Macrophage RAW264.7 cells were incubated with 5 μM PBS in the dark at 37°C for 30 min using the ROS-sensitive probe CellROX Green Reagent, then harvested with 0.05% trypsin-EDTA solution, suspended in fresh medium, and analyzed immediately by flow cytometry.

### Enzyme-linked immunosorbent assay (ELISA)

Malondialdehyde (MDA) and human superoxide dismutase (SOD) antigens were coated on the microplate, and 100 μL of antigen was added to each well, which was then placed at 37°C for 4 h, and the liquid in the wells was discarded. Next, 5% FBS was added for blocking at 37°C for 40 min. After washing 3 times, the diluted sample was added to the microplate wells, 100 μL per well, and was placed at 37°C for 40–60 min. After washing 3 times with washing solution, enzyme-labeled antibodies were added for reaction at 37°C for 60 min. After washing three times, TMB (a chromogenic substrate) was added to terminate the reaction. The OD value was measured at a wavelength of 450 nm with a microplate reader. The concentrations of SOD and MDA in the sample were calculated by plotting a standard curve.

### Experimental mice as well as modeling groups

In this study, 18 female C57BL/6 mice, aged 10–12 weeks and weighing 20–25 g, were selected. The mice were kept in an SPF level laboratory and the state of the mice was observed daily. 5 animals were raised in each cage, no water limit, standard diet, room temperature was maintained at 24~26°C, humidity was 55~60%, 12/12 hours of light time, and mice were kept in SPF laboratories during the experiment. The mice are divided into sham group, model group and LP45 group, with 6 mice in each group. The model group and LP45 group C57BL/6 female mice, after 75% alcohol disinfection injection site, 3% isoflurane anesthesia, after their sluggishness, back skin preparation, disinfection of the surgical site, from the back near the kidney area on both sides of the longitudinal incision, incision fascia to separate the muscle and peritoneum, find the pink ovarian tissue attached to the adipose tissue, complete removal of the ovarian tissue on both sides, the remaining Sham group mice, then remove a small piece of adipose tissue next to the ovary, surgical needles and sutures layer by layer, gentamicin disinfection. The wound was continuously applied with gentamicin for three days after surgery to prevent surgical infection. X-rays are then performed. The animal experiments in this study have been approved by the Laboratory Animal Ethics Committee of Hebei North University. Ethics Number: 2022011805.

### HE staining

Tissue sections were deparaffinized and stained in hematoxylin solution for several minutes. Hematoxylin for 5 min, hydrochloric acid alcohol acidification for 2 s, ammonia anti-blue for 30 s, then rinse in tap water for 1 hour, then briefly rinse in distilled water. After dehydration in alcohol, the sections were stained with eosin solution for 3 minutes, dehydrated in alcohol, cleared in xylene, mounted with neutral gum, and observed under a light microscope.

### Statistical analysis

SPSS 25.0 and GraphPad were used for data analysis and processing. Measurement data were expressed as mean ± standard deviation $\left(\bar{x}\pm s\right)$ and analyzed by chi-square test for comparison among multiple groups, and independent samples *t*-test for comparison between two groups. *p* < 0.05 was considered statistically significant. The independent sample *t*-test was used for comparison between the two groups. *p* < 0.05 was considered statistically significant.

## RESULTS

### Expressions of oxidative stress-related proteins in macrophages and ROS fluorescent probe

**Figure 1:**
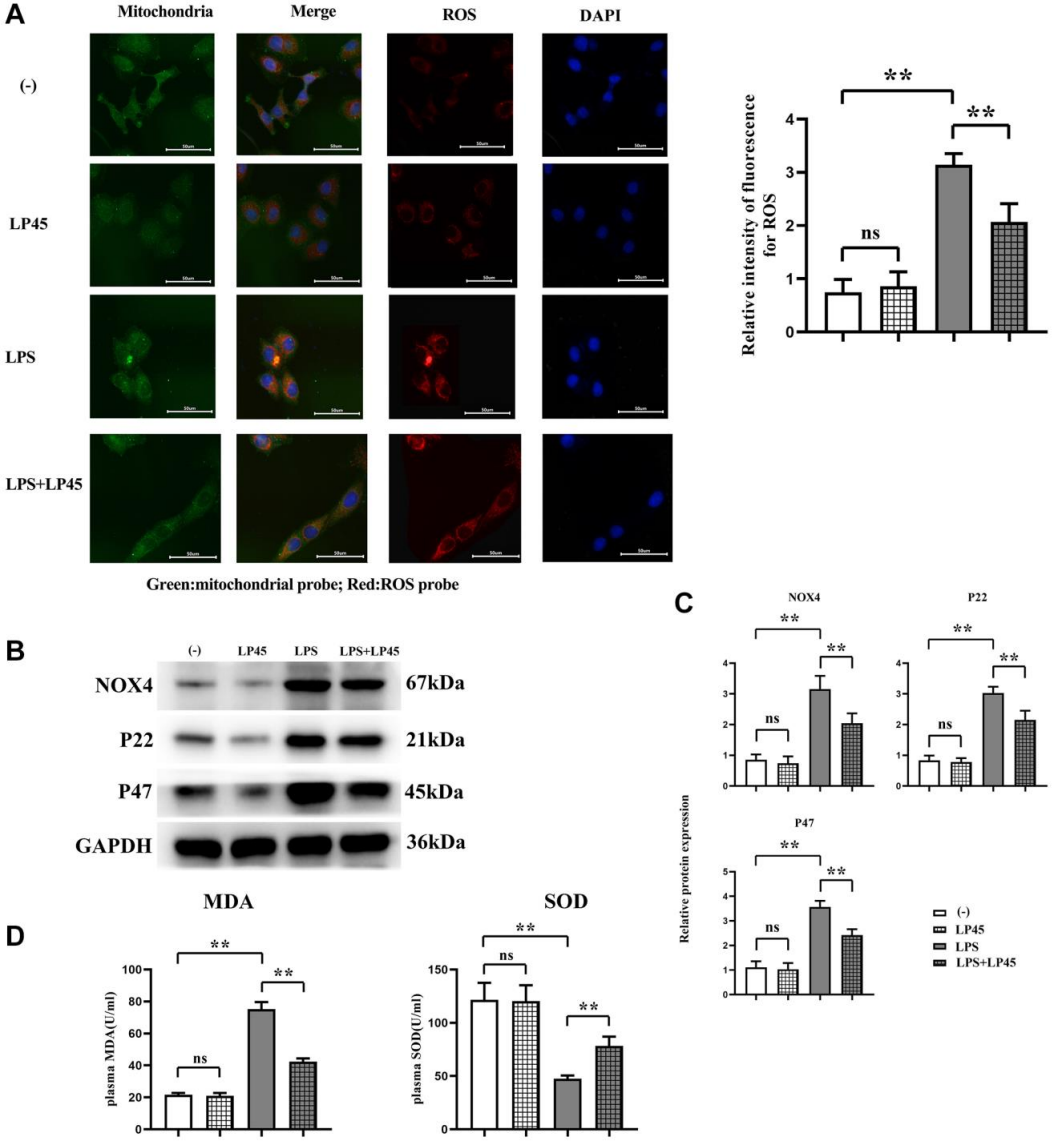
**Detection of oxidative stress level and protein expressions of NOX4, P22 and P47 in macrophages via immunofluorescence and Western blotting, respectively, and detection of MDA and SOD concentrations via ELISA.** (**A**) Detection of oxidative stress level of macrophages in each group via immunofluorescence and relative fluorescence intensity of ROS in LPS group and LPS + LP45 group. (**B**) Detection of protein expressions of NOX4, P22 and P47 in the four groups of macrophages via Western blotting. (**C**) Protein expressions of NOX4, P22 and P47 in macrophages. (**D**) Detection of MDA and SOD concentrations via ELISA. ^**^p < 0.05, Abbreviation: ns: no statistically significant difference.

The results of ROS fluorescent probe showed that there was no significant difference in the relative fluorescence intensity of ROS between the blank control group and the LP45 group. Compared with the blank control group, the relative fluorescence intensity of ROS in the LPS group was significantly increased. However, after the addition of LP45 in the LPS group, the ROS was significantly reduced, indicating that the oxidative stress was significantly reduced. The results of Western blotting showed that there was no significant difference in the relative protein expression levels of NOX4, P22 and P47 between the blank control group and the LP45 group. Compared with the blank control group, the protein expressions of NOX4, P22 and P47 in the LPS group were significantly increased. However, the protein expressions of NOX4, P22 and P47 in the LPS group decreased significantly after the addition of LP45. The ELISA results showed that there was no significant difference in the concentrations of MDA and SOD between the blank control group and the LP45 group. Compared with the blank control group, MDA was significantly increased and SOD was significantly decreased in the LPS group. LP45 significantly increased the concentration of SOD in LPS and decreased the concentration of MDA (Figure 1).

### Expressions of inflammatory cytokine-related proteins in macrophages

**Figure 2:**
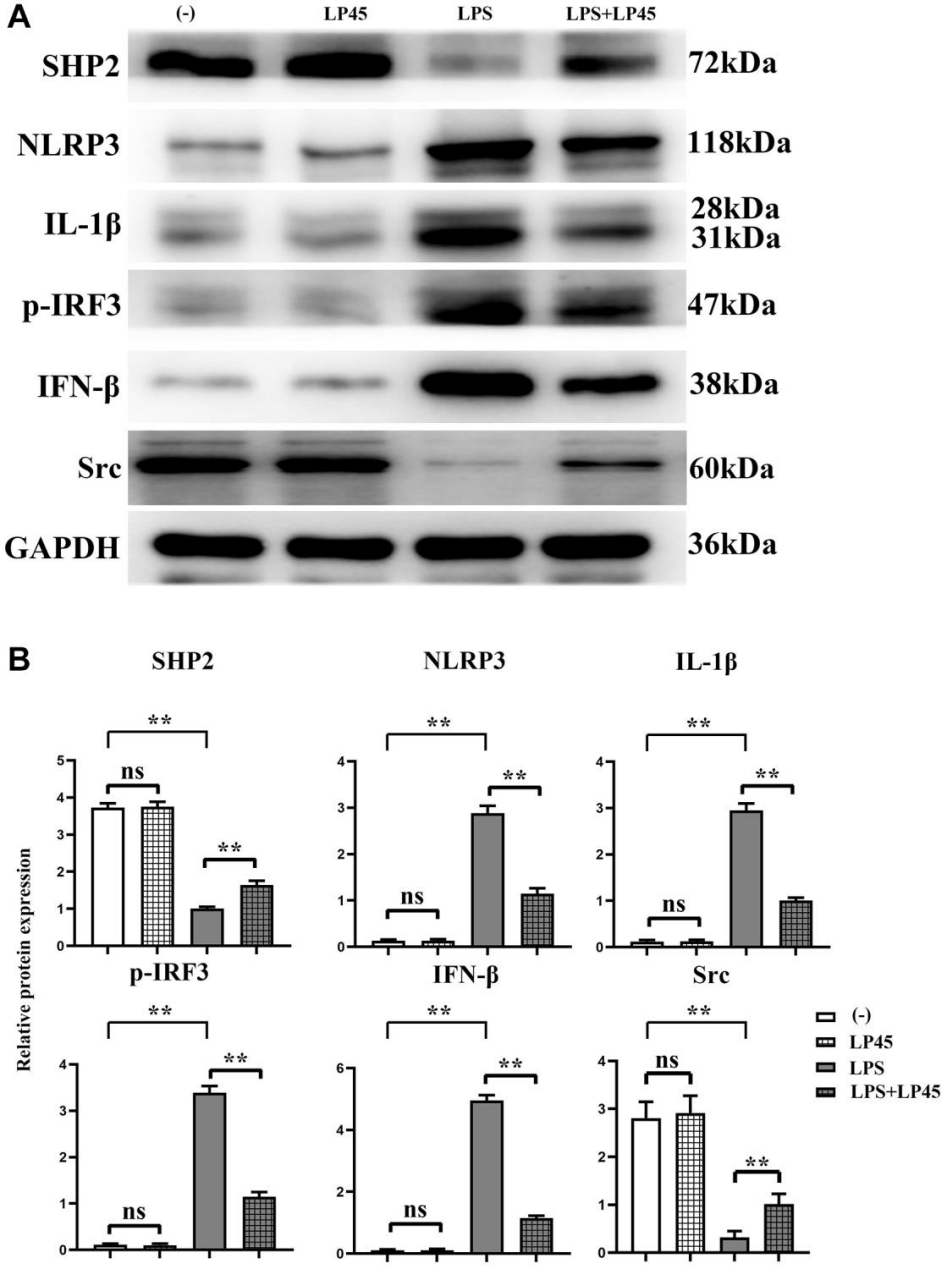
**Detection of expressions of inflammatory cytokine-related proteins in macrophages via Western blotting.** LP45 can activate SHP2 and thus inhibit the expression of these inflammatory cytokines. (**A**) Detection of expressions of inflammatory cytokine-related proteins in macrophages via Western blotting. (**B**) Expression levels of inflammatory cytokine-related proteins in macrophages. ^**^p < 0.05, Abbreviation: ns: no statistically significant difference.

The results of Western blotting showed that there was no significant difference in the relative protein expressions of SHP2, Src, NLRP3, IL-1β, p-IRF3 and IFN-β between the blank control group and the LP45 group. Compared with the blank control group, the protein expressions of NLRP3, IL-1β, p-IRF3 and IFN-β in the LPS group were significantly increased, and the relative protein expressions of SHP2 and Src were significantly decreased. However, compared with the LPS group, the relative protein expressions of NLRP3, IL-1β, p-IRF3 and IFN-β in the LPS+LP45 group were significantly decreased, and the relative protein expressions of SHP2 and Src were significantly increased (Figure 2).

### Expressions of relevant proteins in osteoblasts and RANKL immunofluorescence staining

**Figure 3:**
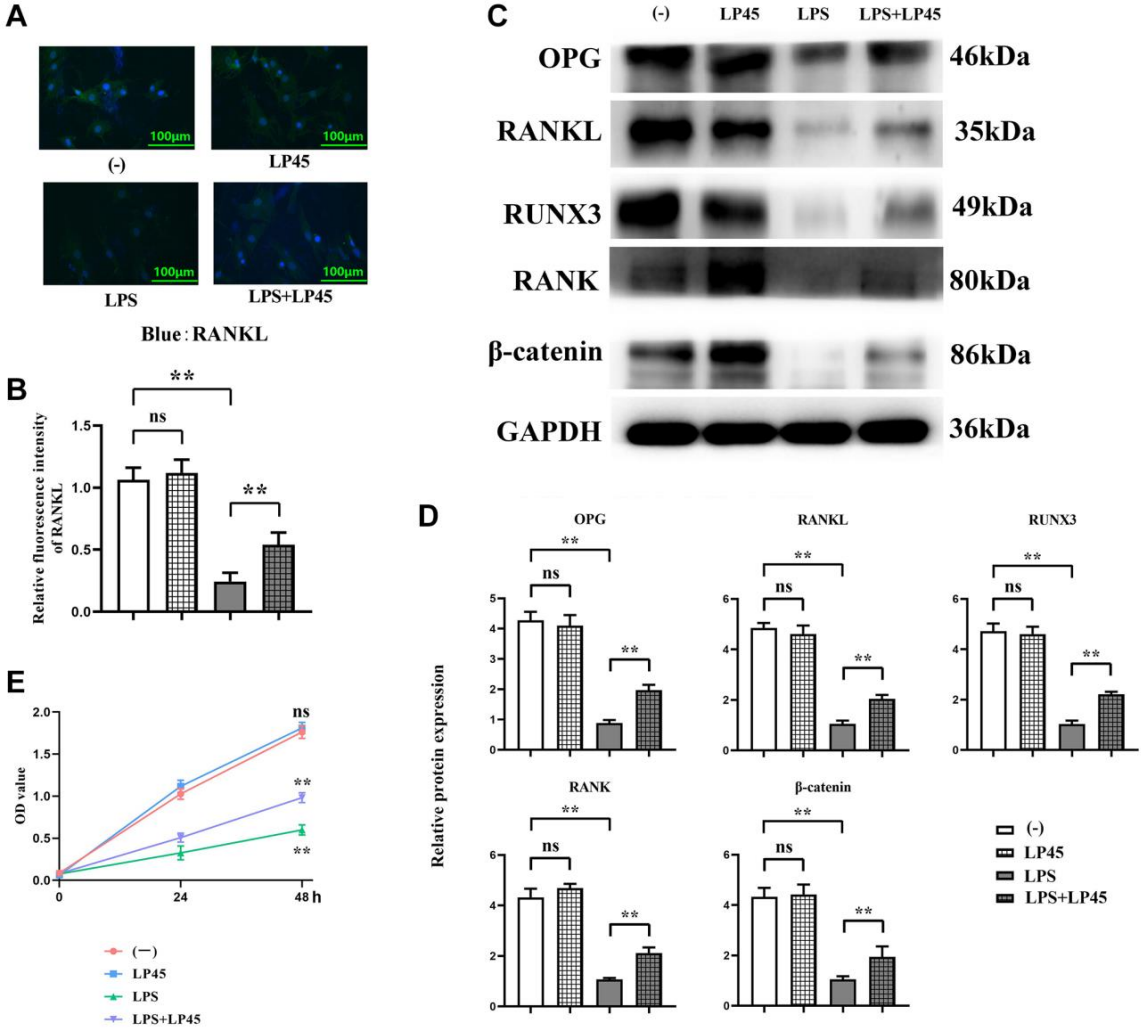
**Detection of expressions of relevant proteins in osteoblasts via immunofluorescence and Western blotting.** LP45 promotes osteoblast differentiation by regulating histone proteases in macrophages and secreting exosomes to osteoblasts. (**A**) Detection of RANKL expression in osteoblasts via immunofluorescence. (**B**) RANKL expression in osteoblasts of each group. (**C**) Detection of expressions of relevant proteins in osteoblasts via Western blotting. (**D**) Expression levels of relevant proteins in osteoblasts. (**E**) CCK8 experimental results. ^**^p < 0.05, Abbreviation: ns: no statistically significant difference.

After glucocorticoids were pretreated with osteoblasts and co-cultured with macrophages, the results of immunofluorescence staining showed that there was no significant difference in the relative fluorescence intensity of RANKL between the blank control group and the LP45 group. Compared with the blank control group, the relative fluorescence intensity of RANKL in the LPS group was significantly reduced. However, compared with the LPS group, the relative fluorescence intensity of RANKL in the LPS+LP45 group was significantly increased. Western blotting results showed that there was no significant difference in the relative protein expressions of OPG, RANKL, RUNX3, RANK and β-catenin between the blank control group and the LP45 group. Compared with the blank control group, the relative protein expressions of OPG, RANKL, RUNX3, RANK and β-catenin in the LPS group were significantly reduced. However, compared with the LPS group, the relative protein expressions of OPG, RANKL, RUNX3, RANK and β-catenin in the LPS+LP45 group were significantly increased. The results of CCK8 showed that there was no significant difference in OD values between the blank control group and the LP45 group at 48 h. Compared with the blank control group, the OD value of the LPS group was significantly decreased, while the OD value of the LPS+LP45 group was significantly increased compared with the LPS group (Figure 3).

### Expressions of relevant proteins in osteoclasts

**Figure 4:**
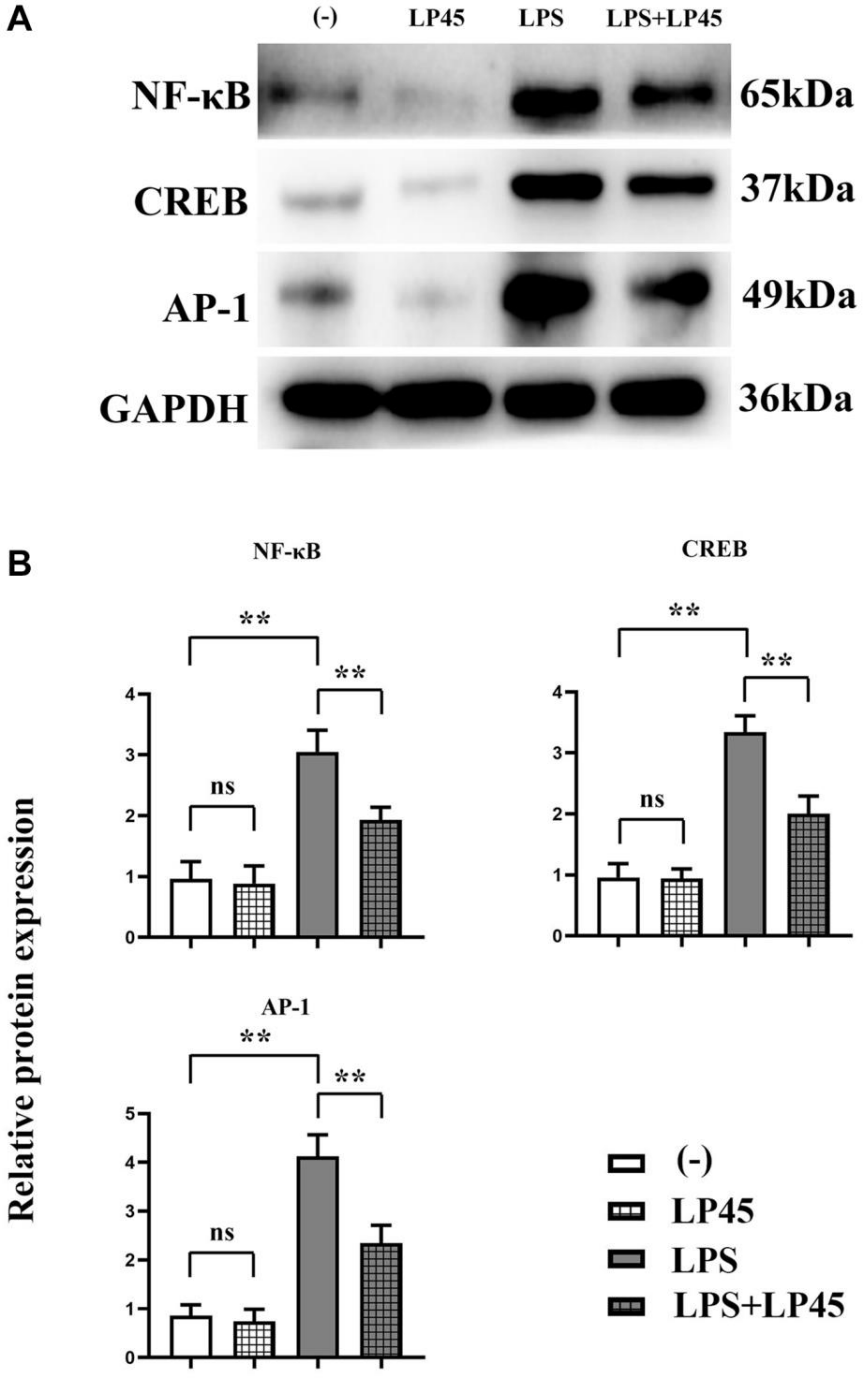
**Detection of expressions of relevant proteins in osteoclasts via Western blotting.** LP45 inhibits osteoclast formation by modulating histone proteases in macrophages and secreting exosomes to osteoclasts. (**A**) Detection of expressions of relevant proteins in osteoclasts via Western blotting. (**B**) Expression levels of relevant proteins in osteoclasts. ^**^p < 0.05, Abbreviation: ns: no statistically significant difference.

The results of Western blotting showed that there was no significant difference in the relative protein expressions of NF-κB, CREB and AP-1 between the blank control group and the LP45 group. Compared with the blank control group, the relative protein expressions of NF-κB, CREB and AP-1 in the LPS group were significantly increased. However, compared with the LPS group, the relative protein expressions of NF-κB, CREB and AP-1 in the LPS+LP45 group were significantly reduced (Figure 4).

### Expressions of relevant proteins in SHP2-knockout macrophages

**Figure 5:**
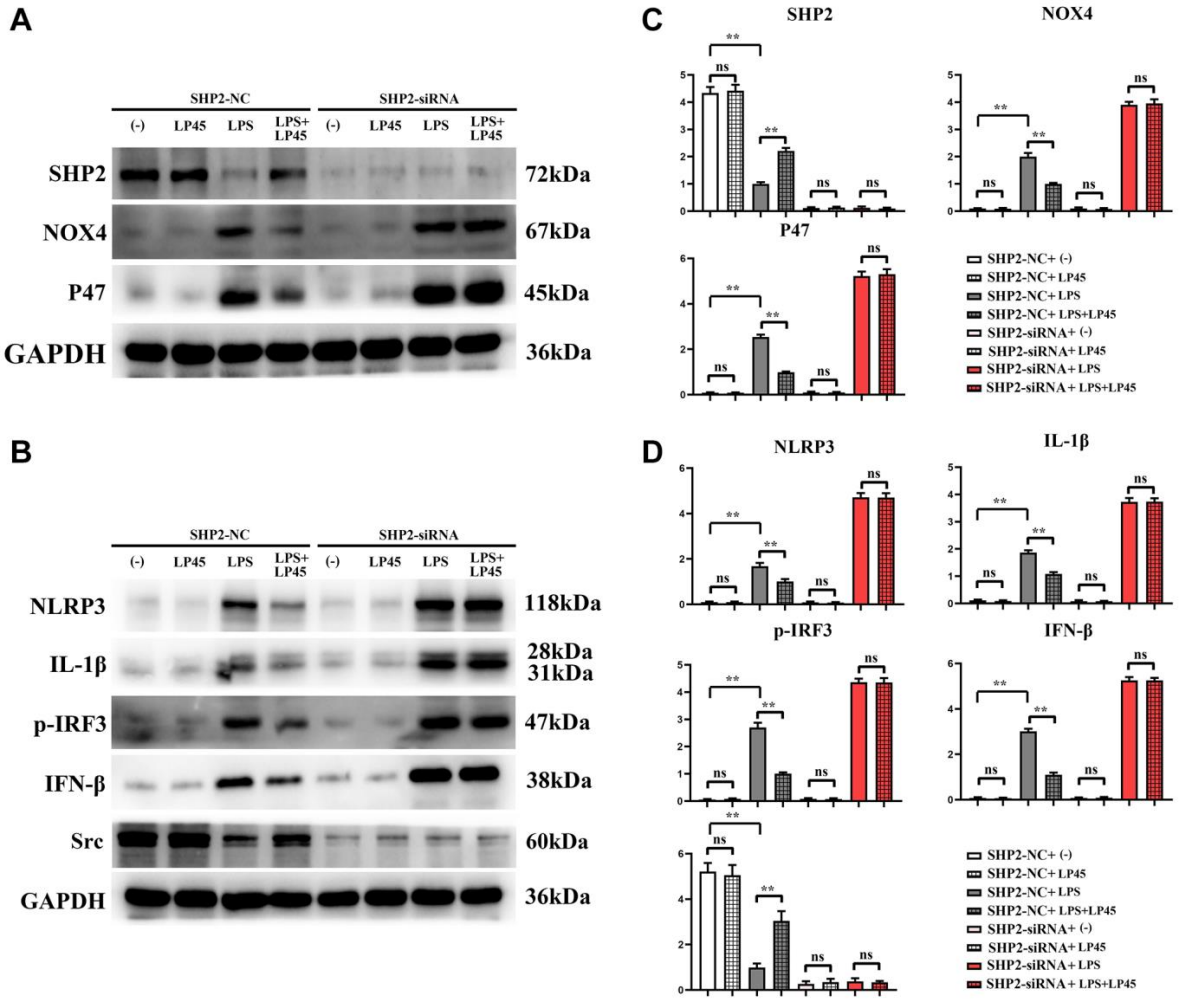
**Detection of expressions of relevant proteins in macrophages with and without knockout of SHP2 via Western blotting.** LP45 can exert its anti-oxidative stress effect to activate SHP2, thus modulating inflammatory cytokine expressions to regulate osteoblast and osteoclast differentiation. (**A**) Western blot was used to detect the expression of SHP2, NOX4 and P47 in SHP2-knockout macrophages. (**B**) Western blot was used to detect the expressions of NLRP3, IL-1β, p-IRF3, IFN-β and Src in SHP2-knockout macrophages. (**C**) Expression statistics of SHP2, NOX4 and P47. (**D**) Expression statistics of NLRP3, IL-1β, p-IRF3, IFN-β and Src. ^**^p < 0.05, Abbreviation: ns: no statistically significant difference.

The results of Western blotting showed that there was no significant difference in the relative protein expressions of SHP2, Src, NOX4, P47, NLRP3, IL-1β, p-IRF3 and IFN-β between the blank control group and the LP45 group. Compared with the blank control group, the relative protein expressions of NOX4, P47, NLRP3, IL-1β, p-IRF3 and IFN-β in the LPS group were significantly increased, and the relative protein expressions of SHP2 and Src were significantly decreased. However, compared with the LPS group, the relative protein expressions of NOX4, P47, NLRP3, IL-1β, p-IRF3 and IFN-β in the LPS+LP45 group were significantly decreased, and the relative protein expressions of SHP2 and Src were significantly increased. However, after transfection with SHP2-siRNA, the relative protein expressions of SHP2 and Src in the four groups were significantly reduced and there was no significant difference. There was no change in the relative protein expressions of NOX4, P47, NLRP3, IL-1β, p-IRF3 and IFN-β in the blank control group and LP45 group, while the relative protein expressions of NOX4, P47, NLRP3, IL-1β, p-IRF3 and IFN-β were significantly increased and the significant differences were eliminated between the LPS group and the LPS+LP45 group (Figure 5).

### LP45 can inhibit the progression of osteoporosis

**Figure 6:**
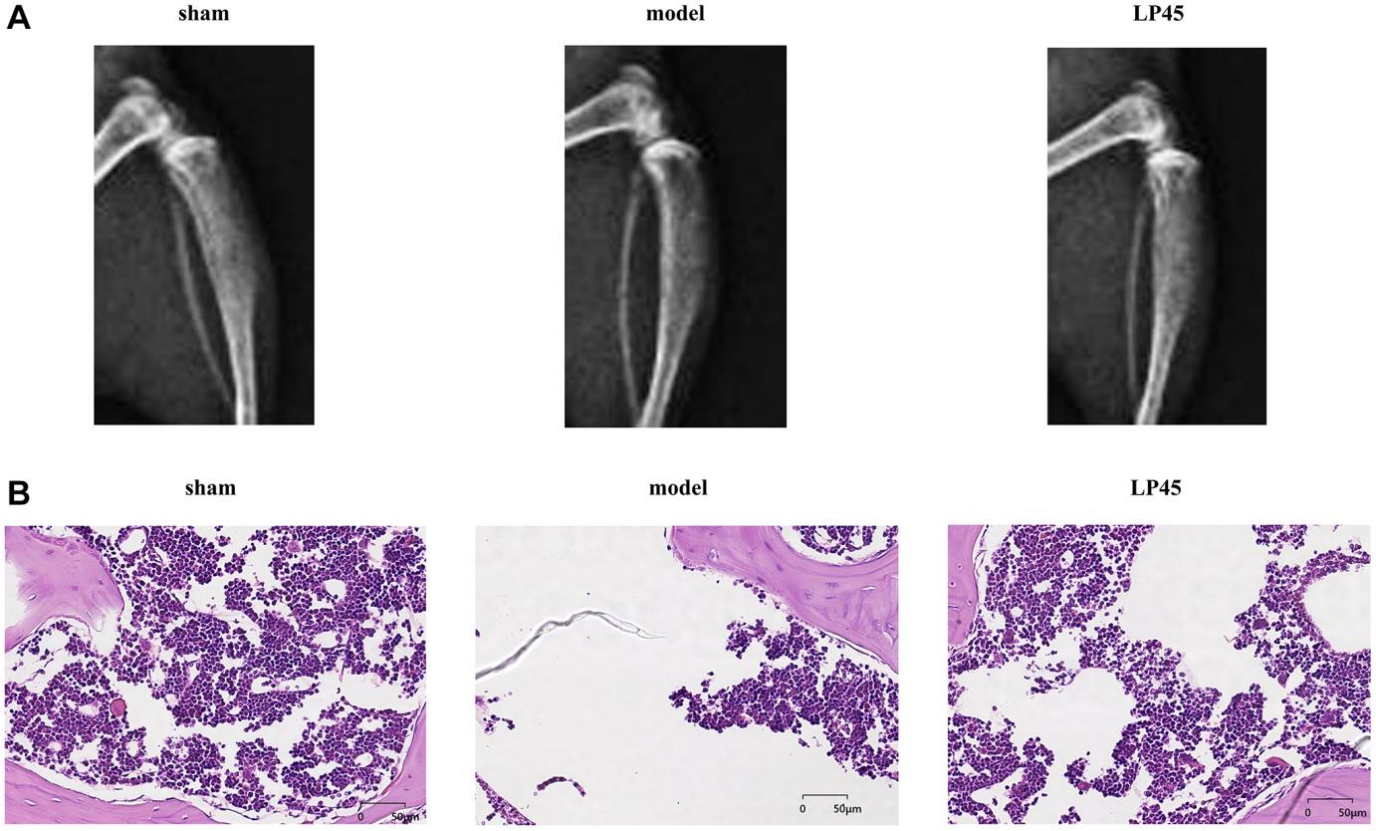
**LP45 can inhibit the progression of osteoporosis.** (**A**) Mice X-ray results; (**B**) HE staining results. ^**^p < 0.05.

**Figure 7:**
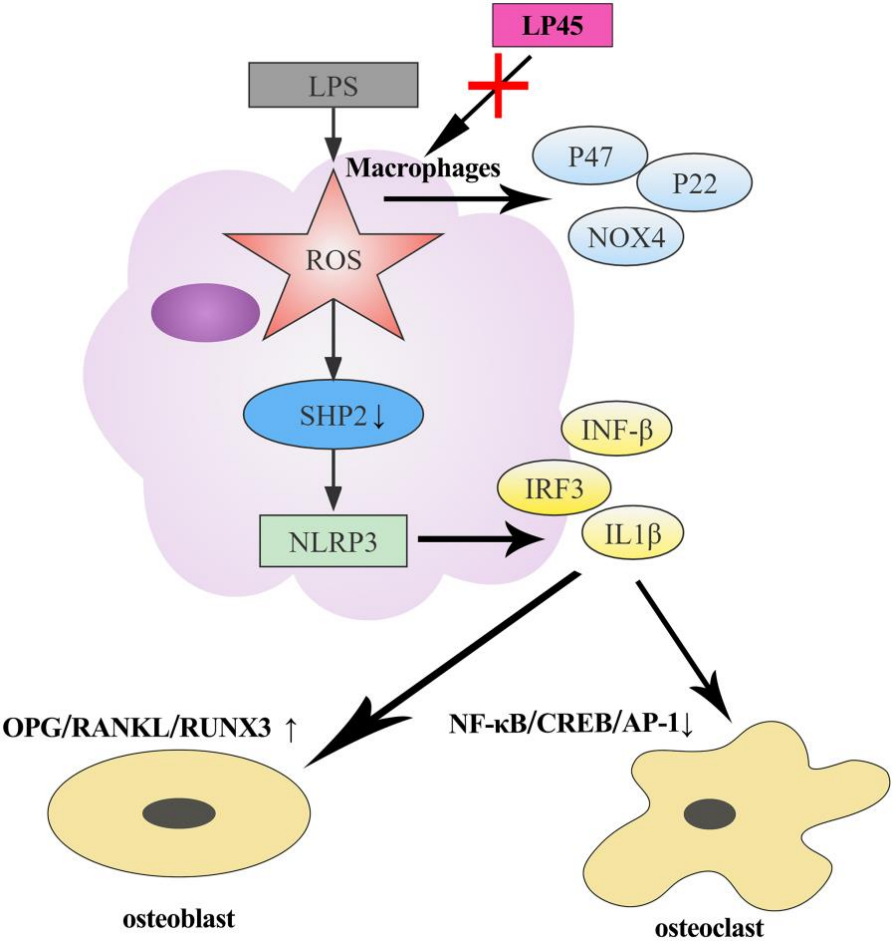
Schematic diagram of LP45 activating SHP2 through inhibition of oxidative stress to regulate osteoblast and osteoclast differentiation.

We observed the effect of LP45 on osteoporosis by X-ray and HE staining. The results showed that the trabecular bones in the Sham group were neatly arranged, had good continuity, were relatively full in structure, and had complete morphological structure. The number of trabecular bones in the model group was scarce, the structure was uneven, the connection was poor, the fracture was broken, the gap widened, and the cavities caused by the fracture and absorption of trabecular bones were visible. However, the number of trabecular bones in the LP45 group was relatively large, thick and full, the gap became smaller, the arrangement was neat, and the continuity of the trabecular bone was better (Figure 6). To sum up, LP45 can exert its antioxidant effect by inhibiting the production of oxidative stress to activate the SHP2 signaling pathway, thus promoting osteoblast differentiation and repressing osteoclast formation to maintain bone homeostasis and improve bone metabolism (Figure 7).

## DISCUSSION

The occurrence of osteoporosis is mainly attributed to the disruption of the dynamic balance of bone reconstruction [22], and oxidative stress is a major player in bone reconstruction and homeostasis [23]. Maintaining osteoblast-osteoclast balance is vital for treating osteoporosis. Immune cells interact with osteoblasts and osteoclasts through cell-cell contacts or secretion of exosomes and are closely associated with various inflammatory bone diseases [24]. It has been found that activated macrophages are closely linked to the severity of osteoporosis [25]. Secretion factors are determinants for regulation of macrophages on bone cells, and specific cytokines are released in response to stimulating factors such as LPS, causing cellular damage and oxidative stress [26]. Macrophages have immunomodulatory effects on *in vitro* tissue recovery and osteoblast differentiation under stimulation by benign factors [27], such as the fact that M2 macrophage-secreted OPG stimulates the differentiation and activation of osteoblast precursor cells [28] (e.g., mesenchymal stem cells, MSCs), increases bone mineralization, and promotes bone homeostasis and bone cell regeneration [29]. Probiotics can influence skeletal system homeostasis and regulate osteoblast-osteoclast balance by mediating the immune system, greatly contributing to skeletal reconstruction [30]. In this study, the specific mechanism by which LP45 exerts antioxidant effects and thus regulates macrophages to promote bone homeostasis was investigated in depth.

Probiotics, defined as living microbial dietary supplements that benefit host animals by improving the balance of gut microbiota, have been extensively studied in recent years. Interest in *Lactobacillus plantarum*, in particular, has grown exponentially in recent decades [31]. Liu X found that *Lactobacillus plantarum* HFY15 played a positive role in regulating Wnt/β-catenin and OPG/RANK/RANKL signaling pathways [32]. Additionally, *Lactobacillus plantarum* GKM3 and *Lactobacillus paracasei* GKS6 inhibited osteoporosis in the OVX mice model, promoted osteoblast differentiation and inhibited RANKL-induced osteoclast differentiation via the Bone morphogenetic protein (BMP) and RANKL pathway promote osteoblast differentiation and inhibit RANKL-induced osteoclast differentiation [33]. Therefore, probiotics exert potentially beneficial effects on bone health.

Oxidative stress increases osteoclast production and osteoblast and osteocyte apoptosis, decreases osteoprogenitor differentiation to osteoblast lineages, and reduces osteoblast activity, so suppression of oxidative stress is a key step in preventing or delaying osteoporosis. NOX4 is the main source of ROS [34], and as vital components of NOX, proteins P22 and P47 are crucial in regulating oxidative stress. The results of this study revealed that the protein expressions of NOX4, P22 and P47 were raised in LPS group, and they were notably decreased after addition of LP45. In addition, ELISA results indicated that LP45 significantly increased the concentration of SOD and decreased the concentration of MDA in LPS, implying that LPS can induce oxidative stress, whereas LP45 has an anti-oxidative stress effect. Inflammatory cytokines function as major regulators in bone metabolism, which can disrupt vascular functions, cause bone loss, inhibit bone mineralization and reduce bone strength by directly or indirectly regulating the formation and function of osteoblasts and osteoclasts [35]. SHP2 is a protein highly expressed in cells and is closely associated with multiple diseases [36]. As reported, SHP2 is closely implicated in osteoblast and osteoclast differentiation. The results of this study manifested that LPS group showed increases in protein expressions of IL-1β, NLRP3, IRF3 and INF-β and a marked reduction in SHP2 expression, while they were the opposite after addition of LP45, indicating that LPS can degrade SHP2 and induce the production of inflammatory cytokines, whereas LP45 can exert anti-oxidative stress effects to activate SHP2 and downregulate inflammatory cytokines.

Abnormal differentiation of osteoblasts and osteoclasts may give rise to an imbalance between bone resorption and bone formation, which is the leading cause of osteoporosis. Glucocorticoids may cause reduced bone formation and bone loss by accelerating osteoblast and osteocyte apoptosis, resulting in the occurrence and progression of osteoporosis [1]. The OPG/RANKL/RANK signaling pathway and the classical NF-κB signaling pathway are pivotal signaling pathways that control osteoclast bone resorption and osteoblast bone formation [37]. Regulating OPG, NF-κB and RANKL expressions by inflammatory cytokines is identified to be a key factor for osteoblast and osteoclast differentiation [38]. The results of this study showed that after the co-culture of macrophages and osteoblasts, the expression levels of IL-1β, NLRP3, IRF3 and INF-β were increased in LPS group, but they were significantly inhibited by the addition of LP45, signifying that LP45 can downregulate the level of inflammatory cytokines and activate osteoblast-related signaling pathways.

Modulating SHP2 and SHP2-related signaling pathways can potentially contribute to bone development and treatment of degenerative diseases, and SHP2 acts as a vital regulator in osteoblast and osteoclast formation [28]. Timothy J Bauler found that SHP2-knockout adult mice have disturbed osteoblast differentiation *in vivo* and present with bone structural abnormalities, such as kyphosis, scoliosis, and osteosclerosis [39]. Yi Zhou discovered that SHP2-knockout mice are prone to osteoporosis, which is associated with the massive differentiation of osteoclasts [40]. It was revealed in this study that after addition of LPS to SHP2-knockout macrophages, the levels of oxidative stress-related proteins such as NOX4, P22 and P47 in cells were raised, and the inflammatory cytokines such as IL-1β, NLRP3, IRF3 and INF-β were highly expressed, while they were the opposite after the addition of LP45. To sum up, LP45 can exert its antioxidant effect by inhibiting the production of oxidative stress to activate the SHP2 signaling pathway, thus promoting osteoblast differentiation and repressing osteoclast formation to maintain bone homeostasis and improve bone metabolism, which offers a new idea and approach for the prevention and treatment of osteoporosis.
